# Dr. Wilhelm Herbst’s Visit to America

**Published:** 1886-08

**Authors:** 


### DR. WILHELM HERBST'S VISIT TO AMERICA.

Reported Expressly for the Independent Practitioner.

At a meeting of the First District Dental Society of the State of New York, held in April, 1885, the Society extended an official invitation to Dr. Wm. Herbst, of Bremen, Germany, to come to this country and give clinics. Dr. Herbst sailed from Bremen on the steamer Aller, June 19th, 1886, and reached New York on Monday, June 28, 1886.

At the June meeting, the President, Dr. Wm. Carr, appointed a reception committee to make arrrangements for the official reception of Dr. Herbst, and Drs. C. F. W. Bödecker, Frank Abbott and Wm. H. Atkinson were appointed, together with the President, Dr. Wm. Carr. They decided to give Dr. Herbst a reception and dinner on Friday, July 2d, at Mazzetti's, 867, 6th Avenue. In the meantime, Dr. C. F. W. Bödecker had invited the Professors of Operative Dentistry of every College of the country, as well as a few friends from New York, Brooklyn, Buffalo and other cities, to meet Dr. Herbst at dinner, at Dr. Bödecker's residence, No. 60 East 58th Street, on Thursday, July 1st. The gentlemen present were Dr. Wm. Herbst, of Bremen, Germany; Dr. Richard Schreiter, of Chemnitz, Germany; Dr. H. J. McKellops, St. Louis, Mo.; Dr. F. H. Rehwinkel, Chillicothe, Ohio; Dr. E. T. Darby, Philadelphia; Dr. C. N. Pierce, Philadelphia; Dr. S. H. Guilford, Philadelphia; Dr. R. R. Andrews, Cambridge, Mass.; Dr. F. A. Levy, Orange, N. J.; Dr. C. S. Stockton, Newark, N. J.; Dr. Wm. Carr, New

York; Dr. Frank Abbott, New York; Dr. Wm. H. Atkinson, New York; Dr. A. L. Northrop, New York; Dr. E. A. Bogue, New York; Dr. Wm. H. Dwinelle, New York; Dr. N. W. Kingsley, New York; Dr. Benjamin Lord, New York; Dr. S. G. Perry, New York; Dr. J. B. Littig, New York; Dr. O. E. Hill, Brooklyn; Dr. Chas. D. Cook, Brooklyn; Dr. Wm. Jarvie, Jr., Brooklyn; Dr. W. W. Walker, New York; Dr. Carl Heitzman, New York; Dr. E. P. Brown, Flushing, L. I.; Dr. C. A. Timme, Hoboken, N. J.; Dr. Ch. Degenhardt, New York; and Mr. E. W. Reuling, New York.

After Dr. Bödecker had spoken a few words of welcome to the guests in general, he presented Dr. Herbst to the assembled company. Dr. Wm. Kingsley then welcomed the honored guest of the evening, and Dr. Herbst responded in German, which was translated by Dr. Bödecker.

Dr. Herbst remarked, that he had not come to this country with anything but hard work in view. He was overwhelmed with joy when he found that at last he had met with candid and straightforward men, who would do justice to the child he desired to entrust to them. When this child (the rotation method of filling teeth) was born, his colleagues in Germany pronounced it unfit to live, and condemned before they had examined it. While in this dilemma, he heard of a well-known American doctor, who proposed to come and examine the bantling. This gentleman was Dr. Bödecker. He gave a critical examination of the infant for one week, and finally pronounced it to be a very healthy boy indeed, declaring that no one would be able to kill it in Germany, and he would try to introduce it into this country. How far Dr. Bödecker has kept this promise you all know. I will only state that, without him, the baby would have died. I am not vain in my expectations, but only hope that you, gentlemen, who stand at the head of the dental profession, and whose names are familiar to almost every dentist in the world, will look at my baby. I shall submit it to your judgment, and if found worthy I shall rejoice with you. I will also be very happy if you will accept the training of it. There is another well-known American child, whose father sits here at my right (Dr. Wm. H. Atkinson), and to him the mallet owes paternity. If you do not, therefore, wish to accept my child alone, then I propose to give it in marriage to that of Dr. Atkinson. Let the rotation method and the mallet be united.

Dr. F. H. Rehwinkel responded, saying that he was very glad that the child was alive, and had come to live in this country. He had always taken a very great interest in the baby, and had therefore come all the way from Ohio to make the acquaintance of both father and child. He knew that the latter had been very badly treated in Germany, that the dental profession would not even look at it, but he was confident that the American people would do justice to anyone. He believed the rotation method a good thing, and knew that the dental profession of this country is only too glad to take hold of anything that is new and good, whatever may be its origin.

Dr. H. J. McKellops said that he was one of the veterans of dentistry. He had seen very many clever fellows, and Dr. Wm. Herbst was one of those whom he delighted to honor. He had made the long trip from St. Louis, and was happy to say that he had been more than repaid for the trouble. If he had seen nothing but the exhibition by Dr. Herbst of the making of a golden nerve-broach, he would have been abundantly satisfied.

A clinic was given in the office of Dr. Bödecker, when Dr. Herbst filled (for a dental student) the right upper second bicuspid, which was very badly decayed. The cavity extended into the distal and grinding surfaces. Before Dr. Herbst began to excavate the cavity he adjusted a matrix of German silver, which was made in the following manner: One end of a thin piece of this metal, about No. 32 American gauge, one-quarter of an inch in width and one inch and a half in length, was pressed between the first and second bicuspids, while the other end was placed between the second bicuspid and first molar, encircling the entire second bicuspid. Then a forcep with blunt cutting edges, made expressly for that purpose and somewhat resembling a pair of ordinary flat-nosed plyers, was applied, and with this the piece of German silver was tightly compressed around the tooth. It was then withdrawn and soldered together with soft solder. After this was accomplished the cavity was prepared in the usual manner, the rubber dam applied, and the matrix put back in its former position. The matrix was made before the excavation was commenced, that the walls of the cavity might be cut away to any amount without losing the contour of the original tooth.

After the matrix had been replaced and the cavity disinfected and dried as usual, Dr. Herbst inserted a few pellets of tin against the cervical border of the cavity, and these were followed by large gold cylinders, which were compressed by a piece of cotton loosely placed over them and pressed down by a hand burnisher, and this followed by an engine burnisher. When thus thoroughly compressed the gold was further condensed into every detail of the cavity by a roof-shaped Herbst instrument in the dental engine, after which a fine exploring instrument, No. 5, was used to see whether the gold had been perfectly packed into every part of the cavity, and in this way layer after layer of gold was added until the entire cavity was filled. The gold used was Wolrab's German cylinders, but at the closing of the operation, Wolrab's No. 10 foil was employed, and this was prepared by cutting one sheet into eight strips, and rolling each up loosely between the fingers.

Dr. McKellops examined the filling very carefully, and found it perfect in every respect. He then expressed his gratitude to Dr. Herbst for his generosity in neglecting his practice and family to come over to this country in order to show his inventions.

Dr. Wm. H. Dwinelle spoke as follows: Gentlemen, Dr. McKellops is my inspiration. After what he has said, I cannot but speak. I am willing to put faith in his new experience and his judgment, enough at least to investigate in all fairness this new system of filling teeth. I am willing to accept new ideas from any source, but will lay aside old ones, only when by the severest tests they are bettered by the new. It is folly to assume that the goal of human progress has been reached and that no further improvements or discoveries can be made. This is simple arrogance and assumption; so when ingenious and honest men, like Dr. Herbst, come among us and generously propose to demonstrate the genuineness and practicability of their discoveries, we are bound in all fairness to give them the opportunity to do so, whether we approve of and adopt them or not. In this spirit we welcome Dr. Herbst. Let us hail him as one of our co-workers, a man of genius, as he certainly is; as an inventor; as one of the prophets and poets of our profession, for I claim that the inventor is the prophet and poet of his time. The true poet looks with the vision of inspiration into the future, and with his far-reaching ken anticipates the slow, plodding unfoldings of time. The inventor, too, is often the philanthropist of his day. He often lives before his time, and as frequently is called a fanatic and a dreamer; "but time at last sets all things even," but with many great inventors, not until they have been long in their graves.

We, as a profession, are an army of inventors. We are inventing from morning till night. It is said that "Necessity is the mother of invention." Bless the good old mother that she so continually surrounds us with such stern necessities, and then obliges us to invent ourselves out of them. I think if all of the practical inventions made by dentists from day to day—most of them in passing, soon forgotten—were sent to the patent office, it would be overwhelmed and retire from business.

You know, gentlemen, I have ever been an advocate of finely attenuated instruments for filling teeth, even the ultimate point, and for instruments of such angles as will enable us to reach every remote and hidden wall, and hermetically to seal every frail and delicate joint. I have assumed that complex operations necessarily must be slow operations. I have said that rapid operators are often apt to be poor operators, so I must accept with caution a system which at first sight seems to be in opposition to the one I have so long practiced.

Dr. McKellops states that by the Herbst method you are not only enabled to fill teeth that will withstand all of the tests that the best operation will endure, but that they can be performed with great facility, reducing the time from one-half to one-third. If this is so, it is a "consummation devoutly to be wished."

The fact that Dr. Herbst uses soft and mellow gold for the sealing of the joints of his cavities, is certainly in favor of their being more thoroughly done than it would be with the use of adhesive gold. I have seen many of these showy adhesive foil fillings lately. Some of them were well executed in the center of the mass of gold, but the borders on the joints of the fillings were full of minute holes, which, though small, were sufficient to destroy the teeth, as a small leak will sink the largest ship.

I am also impressed that Dr. Herbst has seemingly developed a new principle in gold, whereby by the continued rotation of proper instruments under pressure, he not only induces adhesion of the constantly accumulating gold, but he seems to enforce and expand it, so to speak, until it reaches and solidifies against the remotest walls, and closes the joints even to their angles. This may be a new principle; at any rate it is worth the study it invites.

Let us try the new system cautiously and conscientiously, and accept its teachings in all fairness. I do not think such old fogies as myself will ever adopt it in full, but I would not be surprised if it would greatly facilitate and supplement our present methods.

All rotary instruments must of necessity be direct in their action, consequently the undercuts, especially those nearest to you, must be tested with appropriate instruments. This, I understand, Dr. Herbst does, and that at all stages of his work he is constantly operating and testing with instruments terminating in a point. This being the case, the Herbst system will no doubt supplement at least, the operations of all of us who become familiar with it. For the sacrifice Dr. Herbst has made in coming three thousand miles to do us good, for his honest and generous endeavors to benefit and exalt our profession, he deserves our gratitude and respect, whether we adopt all of his views or not, and I trust that when he returns home he will have the happy conviction that in no part of the world has he warmer and more and appreciative friends than in America.

On Friday, July 2d, Dr. Herbst gave two clinics, when he filled with gold, for Miss —— ———, the upper lateral on the mesial surface in nine minutes, and the left upper central in the distal surface in six minutes. He then filled for Dr. Wm. H. Atkinson the left upper canine, the cavity involving the entire mesial surface and a part of the cutting edge. The cavity was very large, and was filled more than twenty years ago with oxy-chloride of zinc by Dr. Butler, and the pulp was then capped. This was found to be alive, but evidently had retracted, as secondary dentine was discovered in the upper part of the pulp chamber. Before the cavity was excavated Dr. Herbst bent a German silver loop matrix around the tooth, similar to the one employed the day previous for the bicuspid, and after the tooth had been prepared, the rubber dam applied, and the matrix readjusted, filling was commenced. The cervical portion of the cavity was filled with a thin layer of tin, and this was followed by Wolrab's gold cylinders. In the last layers of gold, No. 10 foil was made use of. Each layer was critically examined by Dr. H. J. McKellops, of St. Louis; Dr. W. G. A. Bonwill, of Philadelphia; Dr. J. Taft, of Cincinnati; Dr. R. R. Andrews, of Boston; Dr. F. H. Rehwinkel, of Chillicothe; Dr. D. W. Tennison, of New York; and Dr. C. F. W. Bödecker, of New York, and found perfect in every respect. When the operation was completed, the patient expressed his thanks to Dr. Herbst, and his gratification at the exemplification of the new method.

THE FIRST DISTRICT SOCIETY DINNER.

On the evening of July 2d, the members of the First District Dental Society of New York, with invited guests, met at Mazzetti's, where a sumptuous repast awaited them. The occasion was a brilliant one, remarkable for the eloquent speeches, and the witty remarks made by some of the prominent men in the profession, all expressive of their good feeling toward Dr. Herbst, and of their pleasure in having him in their midst. The President, Dr. William Carr, made the opening address, and spoke as follows:

We are congregated here to-night to meet one of Germany's ablest dentists, Dr. Herbst. For years he has pursued a course of investigation and made a series of experiments, principally in the operative department, in order to ascertain the best method of filling teeth. The one which he utilized and adopted is known as the Herbst method, and it is more or less familiar to every dentist of this society. He has come here for the sole purpose of demonstrating his theory of filling teeth, and we assure him that in all societies he will have every opportunity, and will find an appreciative audience. We welcome him as a brother, and we trust that his sojourn amongst us will be a pleasant one.

Dr. E. Parmly Brown said that, as the gentlemen were all so exceedingly modest as to hesitate about speaking, he would take the initiative, and announce that Dr. Atkinson had one of his eye-teeth filled by Dr. Herbst, and it was declared by him to be a marked improvement, and worthy of the notice of all the profession. Dr. Herbst responded in his mother tongue, being kindly interpreted by Dr. Bödecker. He said:

My honored colleagues and gentlemen of the First District Dental Society of New York, when the steamer in which I arrived in this country entered the port of New York I literally trembled, little dreaming or having any anticipation of the generous reception in store for me; I came to this country to show a few little things I have discovered, and you will allow me to take this occasion to thank you for the great pleasure you have given me. It is a well-known fact that the profession of this country is far ahead of any known in the world, and I have been stimulated to see whether I could not do the same work, using different methods. I do not know how to express my thanks to you, for I do not believe that I have earned all that you are doing for me. I feel grateful for anything and everything that has been done in my behalf.

Before me, in my mind's eye, I see such men as Drs. Webb, Varney and Barnum. Dr. Barnum was one of your own members. I bring from every dental society of Germany a wreath in memory of the late Dr. Barnum. Every society of the German Empire is represented here to-night. In the first instance, there is the Central Verein of Germany, of Schleswig Holstein, Saxony and others, There is a ribbon from the Society of Frankfort, the society which first indorsed my invention.

A laurel wreath, from which were suspended various hued ribbons representing the different societies was brought to view, and Dr. Atkinson was requested to accept this token in behalf of the First District Dental Society, and he replied accordingly:

I accept with gratitude and thanks for our society this noble recognition of our departed brother, S. C. Barnum, who, in the generosity of his nature, bestowed this great boon. This is an occasion on which we have a right to congratulate and felicitate ourselves on the recognition of one of our own members, and of the benefit which he bestowed upon his profession and humanity by his inventions. It speaks the honesty of the German mind, and the German nationalities. This should encourage us all to work earnestly and honestly that we may arrive to honor in our calling. What have we here to-night before us ? The Empire State as represented in our profession, and represented by its First District Dental Association. I indorse the idea of acknowledging merit always. We should accept it with gratitude and thanks. I would like to tax your patience a little further in acknowledging the uprightness and straightforwardness of the gentleman whom we meet here to-night. I would like to express the admiration I feel for the manliness of Dr. Brown in acknowledging the good which he has seen, even though it be in the face of his previously expresssd prejudice. I would like to speak of the operation in my own mouth, of the directness with which it was performed, and to assure you that this rotation method is in accord with the principles of the laws of nature.

Now, I want to give a little word of exhortation. I am proud to-night of the dental profession of the United States, and especially of you, my brothers, who sometimes get dissatisfied with the maternity and fraternity which I have preached to you. Here is a man who has been inspired from the same high source, and who has discovered and made known to us the only true way to fill teeth. Let me close by offering the following resolution:

*Resolved*—That this society, on behalf of and in humble commemoration of the late Dr. S. C. Barnum, our beloved and honored member, accept these mementoes from the different German dental societies with many thanks.

Dr. Taft, of Cincinnati, said—I am not so well prepared to speak as those who have just been heard. I am greatly gratified to meet our brother from abroad. We have heard many accounts of him, and I desire to express my gratification and pleasure at seeing an exemplification of his method of operating. I desire to extend my hand to this brother, because it is the first exemplification, the first practical fact that has been exhibited to us by our brethren from other countries. We have not mingled with them as frequently as we ought to have done, and I accept this as an indication of better days in this respect. A profession of Germany, of England, and of France! It will be the profession of the world, when it will be exemplified in character; when that which they have shown to us shall be accepted and improved by us. I feel happy to meet this brother from the other side who comes to teach us something, for I honor anyone who can teach Americans anything. I rejoice to say to him that he can return home with a laurel about his brow that no one else has worn. He has brought to us this system, saying, "This is yours; and you can have it." Would that we had all acted in this respect for the profession. A few have manifested this liberal spirit amongst us, but it is sad to think how few. I hope and look forward to the time when there shall be more mingling together of the professions. The great object in our life is to give to others. What an encouragement when that shall have been carried out to the ultimate; what a grand and glorious profession we will have. I trust that Dr. Herbst will feel on his return home that he has not come in vain, but that he has done great good. We ought certainly to tender him some mete of praise, and I hope that we shall not only know Dr. Herbst, but many of his confreres.

Dr. Rehwinkel—I scarcely know how to express my thanks tonight. My old and beloved friend, Dr. Taft, has said something which I think I can augment a little. In a private conversation with Dr. Herbst, he told me that all Germany was indebted to American genius in every branch of inventive enterprise. That in trying this mode of filling teeth he came to the conclusion that this was the proper method, and felt his way cautiously along, going from one operation to another, until he could proceed with any ordinary operation. But when a brother came to him from America and told him that this method would never be recognized in America unless it could produce contour fillings, he commenced a series of experiments and to find if it were possible to build up teeth by adding one particle to another, and his expectations were realized. He presented it to the profession of Germany. Some believed it a success, some otherwise. Some praised and others condemned. He went from place to place to demonstrate it, and laid it openly before his brothers in the profession, until gradually, step by step, it gained indorsement in Germany. But this did not satisfy him. He wanted the sanction and support of the entire profession. He felt that to crown the system he must turn to the American profession. Thus Dr. Herbst spoke to me, and to-day I have had the pleasure of seeing him operate. This method requires practice, but there is no mode that does not require the same. Dr. Herbst's utter abandonment of self, his generosity in laying his work before us, deserves recognition, and the dental profession cannot but honor these qualities.

Dr. McKellops—I did not come here to make a speech; I came here to learn. I have seen my friend from Ohio operate according to this system, and I have seen others. But that did not satisfy me. I wanted to see the original, and I have seen him to-night. I am proud of this Society. It has exhibited generosity in extending this recognition, and that is a matter in which we may all feel a pride. I have come from St. Louis to see this thing demonstrated. I have watched it closely, but I must first experiment myself before I pronounce it a success. But I tell you gentlemen, Herbst is a genius; he can make or execute more out of nothing than any man I have ever seen. I go home satisfied, and when I talk to those fellows on the other side of the Republic, they will believe what McKellops says.

Dr. Bogue—It is a pleasure to all of us that Dr. Herbst is with us to-day. One by one the influences that control and influence the profession on either side of the great sea become known to us all, and year by year we are becoming one in mind and in spirit. Extending to Dr. Herbst my best wishes, I earnestly hope for an opportunity to witness one of his operations.

Dr. Abbott—I, like some of my friends, came here not to talk, but to eat and drink; simply to enjoy the festivities. I have not the least idea of saying a word, save to wish Dr. Herbst, our friend and brother, the genius, all the success that he deserves.

Dr. Heitzmann—I am greatly pleased at the honor you confer to-night upon a countryman of mine. I experienced the same just twelve years ago. Dr. Bödecker tried the Herbst method on my teeth, and I found it a decided improvement. The great honor that you to-day accord a foreigner reflects honor on yourselves. He came to see you, not to seek a fortune.

Dr. A. H. Brockway—I can hardly express my astonishment and surprise at being called upon to speak. I regard these gatherings of dentists (the best ones in the profession) from various parts of the country, with pleasure. I look upon the social element of dentistry as something which ought to be cultivated in the very highest degree. It is a grand idea, that of a man who has devised something which he considers beneficial to his fellow men, that he should come from abroad to exhibit it to them. What could have impelled him to do this: Selfishness? No. It is something higher and deeper than that, which influences him. He has the good of his profession at heart, and it is that which brings him to our shore.

Dr. C. A. Timme, in a few words expressed his gratitude, thanking the Society for the honor done his countryman, Dr. Herbst.

Dr. Schreiter also expressed his satisfaction and pleasure at the manner in which his colleague was received. He felt particularly grateful to the fraternity for having accepted the token of gratification for the lamented Dr. Barnum in a manner indicating their thorough appreciation of the act, and furthermore declared that he felt warranted in saying that the German profession is keenly alive to the generosity of the American profession, and of the benefits the German is constantly receiving from the American. It had become almost a matter of natural consequence that nothing would be considered good unless it came from this country, and the German profession feels now that, for the first time, it is able to present something in return. He expressed the desire that the feeling of friendliness would continue.

Dr. James McManus—I have heard considerable about Dr. Herbst's method, and I have read a great deal about him. I had a chance to look at that celebrated tooth in the mouth of Dr. Atkinson, and before I go home I hope I shall have the pleasure of seeing the Doctor operate. All I can say is, that I am very glad indeed to be with you to-night.

Dr. Bödecker—About three years ago Dr. Herbst sent me one of his patients with a letter, requesting me to examine the fillings which had been put in by him, and to say if they were in any way equal to the work done by some of the members of the profession in this country. I examined the fillings very carefully, but did not say anything about them, nor write any letter to Dr. Herbst. I thought it a better plan to go to the Dental Society, and forthwith announced to it that I had seen some work done by a new method, and thought that in it there might be a future. I was not mistaken, because very soon after, gentlemen from Stockholm and Paris noticed and remarked it. I made a trip to Germany to investigate. The German Societies had prohibited Dr. Herbst from speaking of his method, and refused to allow him to give a clinic. Why did they act thus? The German Dental profession, collectively speaking, has no method of practice whatever. As a rule, they cannot produce a filling which would satisfy a student of the first year's term. Two years ago, while I was in Germany, the Verein there permitted Herbst to give a clinic. Some there were who talked a great deal of nonsense about it. I told them at that time that I sincerely believed there was something in the matter which I could indorse, and future trials by myself, as well as by others, have verified this. I was in the house of Dr. Herbst a little more than a week, all the while watching his operations very closely, and with the knowledge I had of the matter, I proposed to the First District Dental Society to extend Dr. Herbst an invitation, which I thought, if officially given from the Society would be most cheerfully accepted, although its acceptance would prove a sacrifice to himself and family. I wrote to him, and received a most courteous reply. He left his home, patients, all to enable us to perceive whatever he had to show. Last year, and two years ago, I saw a great deal of his invention, but I must say that the improvements made between this year and last are wonderful.

I did not believe that he would be able to accomplish the things he has demonstrated in my presence. That is the reason why, up to this time, I have practiced the Herbst method cautiously, not wanting to mislead anybody, either my patients or myself. I have usually finished my operations by the old method, but from the success I have seen and achieved for the last two years, I feel satisfied that good and solid fillings can be made by the Herbst method. It requires a deal more of practice to produce perfection than I have had any idea of. I feel more indebted to Dr. Herbst, on account of having witnessed and practiced more of his method, than anybody in this country, and because I have derived more benefit therefrom.

Dr. Herbst was again prevailed upon to respond. He expressed his regrets that he had understood but little of what had been said, but when it was explained to him he said that had he comprehended all that was mentioned during the time he would have left the room, as more had been expressed than he deserved. He said that he had always honored the First District Dental Society, and had always desired to bring his method before it. He had found that many Germans who had been with him and become really interested in the method, had made as poor or worse gold fillings than they had made with amalgam in the old mode. So far, among those who had given him honest assistance, the foremost was Dr. Bödecker. To him, above all others, did he owe a debt of gratitude. Had it not been for him, the Herbst method would not have become known. He felt truly and deeply grateful to him for having been a sincere advocate. His great ambition had been to bring this method before the First District Dental Society in America, and now he felt satisfied. When he received news from America that this method had really obtained some recognition here, he was content. Nay, more than that; it would be impossible to express his gratification, for it was the first instance in his career in which any body of dentists had acknowledged that there was some method in his madness. He anticipated great pleasure in again seeing those present when he attended the International Medical Congress, and hoped to meet them all next year.

